# How to establish workplace learning in long-term care: results from a World Café dialogue

**DOI:** 10.1186/s12912-022-00999-8

**Published:** 2022-08-29

**Authors:** Merel E. A. van Lierop, Judith M. M. Meijers, Erik van Rossum, Johanna E. R. Rutten, Theresa Thoma-Lürken, Sandra M. G. Zwakhalen

**Affiliations:** 1 Department of Health Services Research, Care and Public Health Research Institute, Maastricht University, Duboisdomein 30, Maastricht, 6229 GT The Netherlands; 2 Living Lab in Ageing and Long-Term Care, Duboisdomein 30, Maastricht, 6229 GT The Netherlands; 3 Zuyderland Medical Center, Zuyderland Care, Dr. H. van der Hoffplein 1, 6162 BG Sittard-Geleen, the Netherlands; 4 Research Centre for Community Care, Academy of Nursing, Zuyd University of Applied Sciences, Heerlen, The Netherlands

**Keywords:** Nursing homes, Community care, Nursing staff, Long-Term care, Learning and improvement climate, Continuous improvement, Workplace learning

## Abstract

**Background:**

As long-term care continues to change, the traditional way of learning for work purposes is no longer sufficient. Long-term care organisations need to become ‘learning organisations’ and facilitate workplace learning for nursing staff teams. Therefore, insight is needed into what conditions are important for establishing workplace learning. The aim and objective of this article is to gain insight into necessary individual, team and organisational conditions for nursing staff to enhance workplace learning in long-term care settings.

**Methods:**

This study is a qualitative explorative study. A World Café method was used to host group dialogues in which participants (*n* = 42) discussed certain questions. Group dialogues were held for the nursing home and community care setting separately due to organisational differences. Nursing staff, experts in workplace learning, educational staff, client representatives and experts in the field of work and organisation in healthcare organisations were invited to a Dutch long-term care organisation to discuss questions of interest. Data were analysed using theme-based content analysis.

**Results:**

Overall themes concerning individual, team and organisational conditions for workplace learning included: facilitating characteristics (e.g. to be given time and room for [team] development); behavioural characteristics (e.g. an open attitude); context and culture (e.g. feeling safe); cooperation and communication (e.g. giving/receiving feedback); and knowledge and skills (e.g. acquiring knowledge from each other). No major differences were found between settings.

**Conclusions:**

By assessing the themes at the individual, team and organisational level regarding nursing staff, the current workplace learning situation, and its possible improvements, can be detected.

## Background

Long-term care provided in the Netherlands by nursing staff is becoming more complex due to the increasing number of older people who are often chronically ill, as well as changing perspectives on the definition of health and ‘good care’ [1, 2]. In addition to these changes in long-term care, another challenge is the contemporary shift ‘from *working and learning* to *working is learning*’ that is taking place, where continuous learning and improvement of care needs to be part of every daily practice [3]. To establish such a shift in learning, demands such as the requirement for an appropriate learning design for the health organisation are necessary. Research and educational institutions should work together to establish such learning designs within care organisations. However, the traditional way of learning for work purposes, such as by attending external trainings to gain knowledge and skills, is no longer sufficient [4]. The transfer of learned knowledge and skills from an external training or other external education method to the workplace is difficult [5]. Long-term care organisations therefore need to become ‘learning organisations’ and facilitate continuous learning and improvement for nursing staff teams at the workplace [4]. This is referred to as *workplace learning* and is expected to result in more effective learning than traditional (classroom) education [4].

Workplace learning is informal learning – sometimes combined with formal learning – which takes place during daily practice with the goal of improving the competencies of employees, enhancing their knowledge and improving quality of care [6]. It aims to create learning opportunities at work, where employees acquire knowledge, skills and attitudes that influence their professional development and therefore influence the organisational performance positively [7]. The importance of workplace learning is acknowledged not only within the organisational or business setting, but also within Dutch vocational education. According to Poortman and Visser (2009), there are two main reasons why workplace learning has become an important component in Dutch vocational education. The first reason is that participants develop skills and share knowledge during workplace learning which are insufficiently obtained during traditional education. Second, the connection between education and professional practice is promoted through workplace learning [8]. Workplace learning is also financially attractive, results in active participation of employees and increases the innovation competencies of professionals in daily practice [9, 10].

However, to facilitate workplace learning for nursing staff teams in long-term care organisations, insight into the conditions for establishing workplace learning in nursing is needed. Within healthcare, research has shown that there are different ways of looking at learning in other settings, such as hospitals [11]. On the one hand, conditions that exist in a workplace with regard to learning are important, and these can include the availability of resources such as manuals, the presence of colleagues who stimulate learning or the presence of a supervisor who supports learning [11]. On the other hand, the focus can be on the atmosphere within the organisation – that is, the culture with regard to learning. Decuyper et al. (2010) have shown similar conditions as predictors of informal team learning and described these predictors at *the level of the individual, the team* and *the organisation*. Decuyper et al. (2010) distinguished these three levels because they are important to manage the continuously changing environment in every modern organisation, and are therefore important for workplace learning. At the level of the individual, examples of conditions for informal team learning include being motivated, flexible and having high self-efficacy. At the team level, examples are team leadership and management or team composition. At the organisational level, organisational strategy and leadership are mentioned. Leaders are important because they should proactively manage team learning and remain constantly involved in the learning process [12].

Several articles mention important conditions for workplace learning in nursing care, such as having a safe team climate, or increasing nurses responsibilities and independence [13, 14]. However, at this point, conditions for the individual, team, and organisational levels for nursing staff working specifically in long-term care (nursing home or community care setting) remain largely unknown. It is important to identify more detailed information about these conditions to be able to operationalise workplace learning in the nursing setting. Therefore, the current study sought to identify the necessary individual, team and organisational conditions for nursing staff to enhance workplace learning within a long-term care setting (nursing home setting and community care). The identification of conditions offers a starting point for long-term care organisations to become ‘learning organisations’ and facilitate workplace learning for nursing staff teams.

## Methods

### Design

We conducted a qualitative exploratory study using a World Café dialogue in which participants discussed necessary conditions for establishing workplace learning in small group discussions [15, 16]. This method is a creative process to promote collaboration and to share knowledge and ideas, which makes it possible to create vivid conversations and actions [17]. In an informal ‘café’ environment, several small groups of participants discussed questions of interest at different tables. All participants shuffled between tables and thus formed new subgroups in which they discussed and built on previously mentioned ideas from the prior subgroup. At the end of the World Café, the ideas and findings from all subgroups and tables were discussed plenary through a large group conversation.

### Participants

This study was embedded within the Living Lab in Ageing and Long-Term Care [18], which is a structural collaboration between multiple long-term care organisations and (vocational and bachelor) educational and research institutions. By connecting research, practice and education, its mission is “to contribute with scientific research to improving i) quality of life of older people and their families; ii) quality of care and iii) quality of work of those working in long-term care. Key working mechanisms are the Linking Pins and interdisciplinary partnership using a team science approach, with great scientific and societal impact” [18]. Study participants were only recruited from this living lab and were included if they were working within a nursing home or community care setting in the following occupations: expert regarding workplace learning (people who are frequently working with workplace learning or who received training on the subject); nursing staff or management working in nursing homes or community care; education expert; or client representation. If participants did not meet these requirements, they were excluded from participation. Purposeful sampling was used for the identification and selection of participants based on occupation and was done by the management of the participating organisations in cooperation with the researchers. We aimed to include 25 participants, and participants were approached and informed through e-mail. If they were interested, they received a save-the-date and invitation with additional information about the study. Participants were asked to register and approve participation by responding via e-mail.

### Procedure for World Café and data collection

**Fig. 1:**
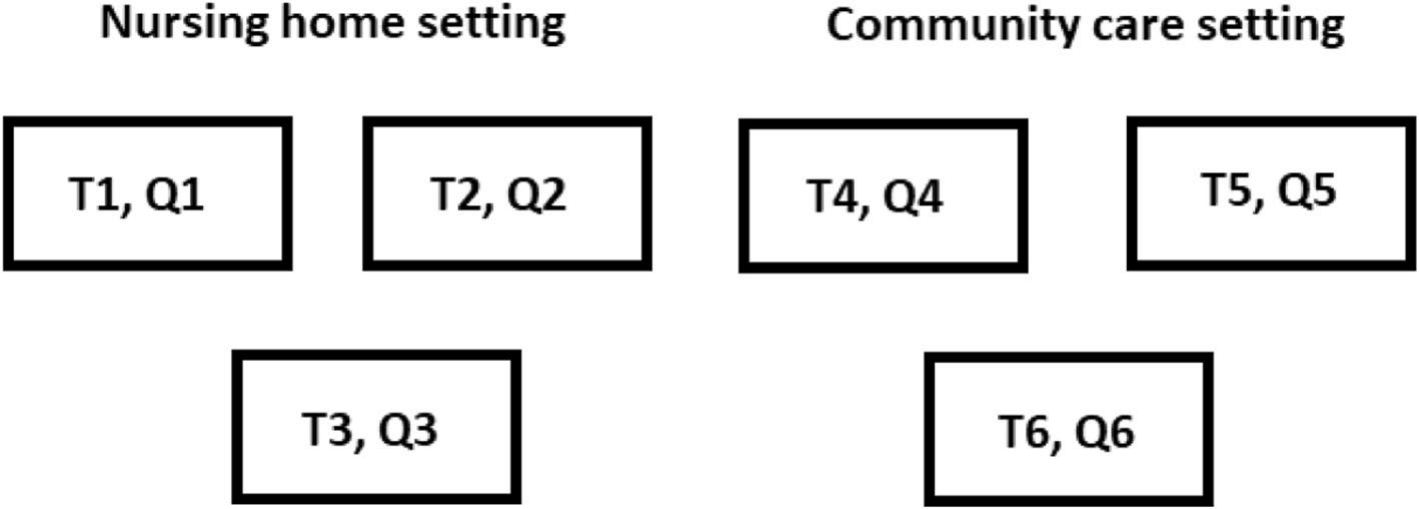
Visualisation of tables (T) with corresponding questions (Q) (see also Table 1)

The World Café was held in September 2019 at of one of the participating long-term organisations. At the beginning of the World Café meeting, participants received an informed consent form and a questionnaire with demographic questions about gender, age, educational level, organisation and years of employment (in direct care), current position, and setting of employment (nursing home care or community care). All participants completed the documents if they agreed with the terms and conditions. The World Café continued with a presentation by a researcher from the Living-Lab in Ageing and Long-Term Care including the specific procedures and aims of that day. Participants were afterwards allocated to one of six discussion tables. Three out of six tables were appointed to nursing home care participants and three to community care participants, ensuring a mix of occupations at each table. In total, six open questions (three for each setting) were discussed (Table 1; Fig. 1). Participants took part in three rounds of 20 min each. After each round, all participants were allocated to another table where another question was discussed in a different group composition.

**Table 1:** Questions World Café for every specific table

Table number	Question
T1	Q1. What individual conditions or competencies would nursing staff require to be able to establish workplace learning in nursing homes?
T2	Q2. What does nursing staff require from their team to be able to establish workplace learning in nursing homes?
T3	Q3. What does nursing staff require from their care manager and organisation to be able to establish workplace learning in nursing homes?
T4	Q4. What individual conditions or competencies would nursing staff require to be able to establish workplace learning in community care?
T5	Q5. What does nursing staff require from their team to be able to establish workplace learning in community care?
T6	Q6. What does nursing staff require from their care manager and organisation to be able to establish workplace learning in community care?

Each table was chaired by a table host (a researcher (MSc or PhD) from the Living-Lab in Ageing and Long-Term Care). The table host remained sitting at the same table and introduced the main question, led the conversation and made notes on a large tablecloth. Additionally, the table host ensured that audio recordings were made with the consent of the participants. To ensure that the views of all participants were taken into account, every round started with all participants writing down their own ideas about the question addressed at their specific table on a sticky note. These notes were then presented and put on the tablecloth that covered the table. After this first inventory, all participants explained their ideas as presented on the sticky notes. Afterwards, further elaboration took place. The table host wrote down information on the tablecloth.

After each round, the table host took a photo of the tablecloth. At the beginning of every new round, the table hosts summarised the dialogues of the previous rounds to the new group of participants at that table to ensure that there were few repetitions by participants and new information was gathered. More in-depth information was gathered during each round. After all rounds, every table host presented the main findings from their question to the whole group of participants and asked if participants had any additions.

### Data analyses

All notes on the tablecloths from the six tables were coded afterwards through open, axial and selective coding [19]. First, the notes were divided into theme-based categories by the first author to identify common themes within each specific question. Each question discussed at one table was analysed separately. This data analysis was repeated independently by the fourth author to ensure rigor. Discrepancies between the two analysts were afterwards discussed until consensus about all themes was reached. The audio recordings were then used to clarify the results, to get more detailed information about the conditions concerning the three different levels and to check the motivation participants gave during the World Café for certain statements. When notes on the tablecloths needed more detailed background information, audio recordings were also used for clarification. A summary of the findings was sent to all participants for a member-check. In addition to sending this summary, participants were asked if they had any feedback on or additions to the findings.

## Results

### Description of the study population

In total 42 people participated in the World Café. Participants included nursing staff (*N* = 20), experts in workplace learning (*N* = 2), educational staff (*N* = 3), client representatives (*N* = 3) and experts in the field of work and organisation in healthcare organisations (*N* = 14). Insight into participants’ demographic and occupational characteristics is given in Table 2.

**Table 2:** Participants’ characteristics (*n* = 42)

Demographic characteristics	
Age in years (mean/range)	46.1 (21–75)
Gender: Female (n)	29
Educational level	
*Middle-level applied education (n)*	7
*Higher professional education (n)*	*24*
*Scientific education (n)*	*11*
**Occupational characteristics**	
Years of experience in current position (mean/range)	7.6 (0–35)
Years of experience in elderly care (mean/range)	14.7 (0–45)
Working as a direct care professional (n; *n* = 40)	25
Setting (*n* = 37)	
*Nursing home (n)*	*24*
*Community care (n)*	*10*
*Both settings (n)*	*3*

Participants were divided into a nursing home setting group and a community care setting group. Although the results showed some slightly different emphases for certain conditions while comparing both settings due to organisational differences, no major differences were found. Because of the absence of such differences, we describe the results of the nursing home setting and community care setting together, using the division into individual, team and organisational levels as a framework [20].

#### Individual conditions required for workplace learning

**Fig. 2:**
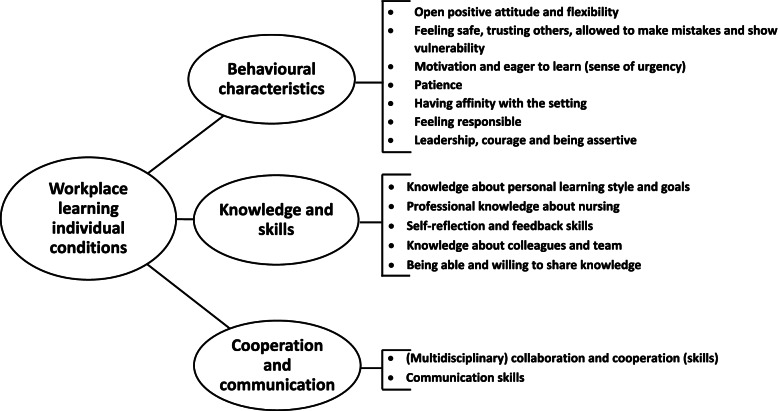
Reported individual conditions for workplace learning

Three main themes were identified within this level: behavioural characteristics, knowledge and skills, and cooperation and communication. Figure 2 shows the conditions per theme.

#### Behavioural characteristics

The results show that an open positive attitude, including being open for change, is considered important for nurses within nursing staff teams to be able to stimulate workplace learning. Being able to feel safe and trusting others were mentioned by participants, and these were linked to the finding that it is important for nursing staff to feel they are allowed to make mistakes and thereby dare to show vulnerability without any consequences.

***Participant (community care setting):*** ‘*And I also think you should not be afraid to make a mistake and not be afraid to speak up if you do. Because you learn from mistakes.’*

Being allowed to make mistakes also comes with a certain level of flexibility to change, which is necessary for sustaining an open attitude. Furthermore, being motivated and eager to learn and change was important according to participants, because being motivated is related to nursing staff feeling a sense of urgency and thus finding it important to apply workplace learning.

***Participant (community care setting):**** ‘But I also think with regard to yourself, if you want to learn you will have to have an open attitude and be interested, of course. So someone should really have to go for it, you should “want” to do an internship in the community care setting. I once saw someone working within a nursing home setting and this person did not want to work there at all. I think in such cases, you do not have a good start and therefore won’t be able to learn anything at all.’*

Additional conditions were patience, having an affinity with the specific setting in which one is working, feeling responsible, taking matters into one’s own hands, leadership, showing courage and being assertive were found necessary individual conditions to be able to establish workplace learning.

#### Knowledge and skills

Participants stated that self knowledge, including knowing one’s own learning style, individual learning goals and professional knowledge about how to perform one’s work as a nurse are crucial for workplace learning. Additionally, the need for knowledge about new concepts such as workplace learning itself was reported.

***Participant (community care setting):*** *‘I think it (workplace learning) is still a relatively unfamiliar concept.’*

To improve this factor of self-knowledge, nurses also need to have proper self-reflection and feedback skills to be able to reflect on their own actions and others. Additionally, for nurses to have insight about (the skills of) their colleagues in their team was found to be of importance, as this can help when it is necessary to seek help at work. Finally, being able and willing to share knowledge was also mentioned, as this also increases the possibility of cooperation with colleagues.

***Participant (nursing home setting):**** ‘You should have experienced colleagues present from all educational levels and there should be a willingness to share (knowledge) with each other. So without thinking: knowledge is power. So I will not tell you anything to make sure you know as much as I do.’*

#### Cooperation and communication

Another result that emerged from this study was that nurses mentioned communication skills would be important for enabling a workplace learning approach.

***Participant (community care setting):*** *‘The right communication skills: people should not be afraid to ask for help … In relation to this there must also be a basis of trust.’*

#### Team conditions required for workplace learning

**Fig. 3:**
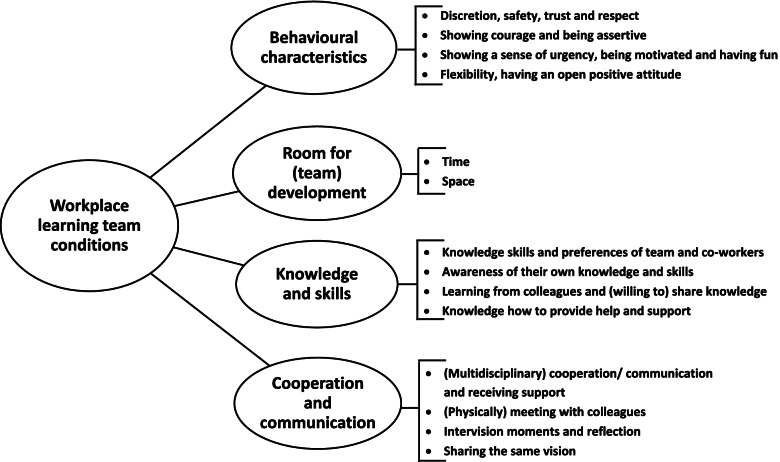
Reported team conditions for workplace learning

Four themes were identified with regard to team conditions: behavioural characteristics, room for development from colleagues, knowledge and skills, and cooperation and communication. Figure 3 shows the conditions that were reported for each theme.

#### Behavioural characteristics

With regard to the behavioural characteristics of colleagues, participants mentioned that it would be important for team members to be discrete, feel safe and that colleagues trust and respect each other. This includes, for example, that colleagues share opinions and dare to show vulnerability without any consequences, but also for colleagues to accept that every individual is different so that everyone will be able to be themselves.

***Participant (nursing home setting):**** ‘...but that I am also allowed to be myself. That I don’t necessarily have to emulate colleagues, but that I am allowed to use not only my own expertise, but also my own learning, in my performance.’*

Another behavioural characteristic included showing courage and being assertive when necessary. Additionally, participants mentioned that it would be important for colleagues to show a sense of urgency for applying workplace learning as an approach, and that they are motivated and understand why it is important to use this approach. This includes flexibility, having an open positive attitude and being open for change. Finally, it was also found to be important for colleagues to have fun while performing their work, as this increases the motivation to work and learn.

#### Room for (team) development

It is regarded as important for nursing staff to be given time and space by colleagues to be able to apply workplace learning.

***Participant (nursing home setting):**** ‘It is often said in one sentence: “take time ... plan it”, and this first sentence is then followed with “there is no time at the moment”. That is done very often … and there will still be no time tomorrow’*

An example with regard to having space included having the freedom to apply workplace learning in the way that works for every individual.

#### Knowledge and skills

Taking into account knowledge and skills, participants mentioned the importance for colleagues to have knowledge about the available skills and preferences of colleagues within their team and of co-workers, but also for colleagues to be aware of their own knowledge and skills. Additionally, participants stated that nursing staff should be able to learn from colleagues. It was therefore added that colleagues need to be willing to help and share knowledge and skills, but they also need to know how to do so. Additionally, these knowledge and skills conditions for workplace learning are as important for older employees as for new employees (or trainees). There is often a gap between older employees and trainees or new employees in terms of being ready to work in a nursing staff team in real life practice. New employees often have less knowledge and skills and need to be trained to work within a certain team.

***Participant (community care setting):**** ‘And (an overview of) the need (of knowledge in a team) of course. You also need to have insight into the need to have certain knowledge in a team ... so you can zoom in on these needs.’*

#### Cooperation and communication

First, to stimulate workplace learning, participants mentioned (multidisciplinary) cooperation and communication of colleagues as being important. This includes receiving help from colleagues during daily work struggles and for colleagues to seek (face-to-face) contact with nurses.

***Participant (community care setting):**** ‘What I experience is that people in nursing home care have to run during work and afterwards they go straight home. And what I miss is that you meet each other (nursing staff), that people come by at the office ... having more contact with each other.’*

Participants also mentioned that, due to the increasing use of technology, actual face-to-face contact with colleagues has decreased and with this, the frequency at which communication takes place has decreased. Additionally, colleagues being able to reflect about themselves, their co-workers and the team was found to be necessary to work with an approach such as workplace learning. Furthermore, for colleagues to make use of interviews and feedback to gain suggestions for improvement from colleagues would be of importance. However, colleagues should also know how to give positive feedback and compliments. Finally, for colleagues to share the same vision, support each other and to communicate in a clear way was found to be important for establishing workplace learning.

### Organisational conditions required for workplace learning

**Fig. 4:**
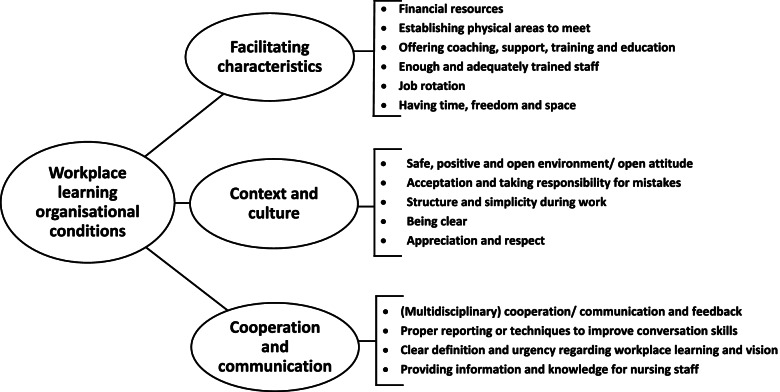
Reported organisational conditions for workplace learning

Finally, organisational conditions were separated into three different themes: facilitating characteristics, context and culture, and cooperation and communication. Figure 4 shows the conditions that were reported per theme.

#### Facilitating characteristics

Organisational facilitating characteristics mentioned by participants to promote the establishment of workplace learning for nursing staff included having financial resources, establishing physical areas to meet (especially for community care nursing staff teams), offering coaching and support, and providing time, freedom and space.

For an organisation to provide space means an investment in the staff through education and training, or to provide time by decreasing the workload. Additionally, sufficient and adequately trained staff who can help implement workplace learning should be provided by the organisation. Organisations should also take into account the different levels of education among nursing staff during the use of a workplace learning approach and the organisation should be aware of the preferences and opinions of the nursing staff. Finally, organisations should make sure that there is enough job rotation so nurses can learn from within different contexts, and enough training and education opportunities for nursing staff should be available. This should include (facilitating factors for) personalised ways of education and training.

***Participant (nursing home setting):**** ‘If I am someone who would like to learn things digitally, decent ICT support should be present. However, if I am someone who learns a lot while sparring with a colleague, then I will need a lot more time.’*

#### Context and culture

According to participants, the organisation should provide a context and culture where workplace learning is stimulated by a safe, positive and open environment within the organisation and nursing teams where nursing staff can feel trust, show vulnerability and where mistakes can be made without any consequences. This includes accepting when things do not go as planned.

***Participant (nursing home setting):**** ‘I think it is also very important for the organisation, ... that nursing staff also feels that they are given the space to learn ... if you, as an employee, do not feel that you are also important, or that you are not looked after ... for example if at some point you are sick or whatever, and at that moment there is no concern about what you are going through or that you may be sick for a while, but that you actually have to resume your work that same day ... then you will not feel seen or heard, and how can you give care to someone else in such a learning climate?’*

An organisation needs to be open for change to establish workplace learning. However, participants also stated that structure and simplicity (‘keeping it simple’) while implementing workplace learning is needed for an approach such as workplace learning to be workable. Participants stated that to improve motivation, the organisation should be clear about why, for example, workplace learning is implemented. Furthermore, other important conditions which should be provided by the organisation were appreciation and respect. Organisations should also take responsibility for mistakes when necessary.

#### Cooperation and communication

To stimulate workplace learning, participants stated that (multidisciplinary) cooperation and communication between organisations and departments is important. Proper reporting or techniques to improve conversation skills could be tools provided by the organisation to improve this cooperation and communication. Additionally, an organisation should be clear about the definition of and urgency for workplace learning, about the vision of the organisation, and should provide enough information and knowledge for nursing staff to work with to gain clarity about the implementation of workplace learning within nursing teams.

***Participant (community care setting):**** ‘Well, I think that in order to learn well, it is important that they (nursing staff) have the right information available at all times. So having the right information when you are in the workplace and in particular the information availability regarding files, policy, agreements, medication, what has been done ... that it is very important.’*

Furthermore, an organisation should facilitate moments for providing positive as well as negative feedback for nursing staff by organising specific moments for reflection. However, employee feedback for the organisation should be taken into account, as participants stated the importance of the organisation listening to employee preferences. Additionally, organisations should know what is happening within nursing staff teams and should stay informed.

## Discussion

This study identified necessary conditions at the individual, team and organisational levels for nursing staff to enhance workplace learning within the long-term care setting. Important conditions indicated were facilitating characteristics like room for (team) development, overall behavioural characteristics such as an open attitude towards workplace learning, context and cultural characteristics like feeling safe to learn and make mistakes, cooperation and communication such as giving feedback, and knowledge and skills like knowing (the situation in) the nursing staff team. Some conditions were similar for all levels, like using proper cooperation and communication. Furthermore, some of the reported conditions come from underlying problems within the specific field of long-term elderly care. An example of these problems is nursing staff being accountable to the higher management of a care organisation for every step taken, which causes the fear of making mistakes and therefore causes a barrier for learning at work. Other conditions (such as giving and receiving cooperative feedback and communication conditions) are applicable for many different kinds of work settings.

An important *facilitating characteristic* our research identified was being given enough time by the organisation to be able to learn in the workplace. Time shortage at work is one of the most common problems mentioned within nursing healthcare, where staff shortages are common [21]. Nursing staff also indicated that the daily care workload was too high, which resulted in no time to invest in learning at work. These findings are in line with a survey of 1573 nursing staff professionals, more than half of whom experienced their work as busy, and over 18% experienced their work as too busy [21]. However, this lack of time could also be caused by having no control over one’s work instead of actual time pressure [22]. For nurses, this is often the case, as they are less able to determine their own pace and order of their tasks. This could be caused by the unpredictability of caregiving, as nurses may, for example, abruptly need to change their work tasks when a crisis on the ward occurs, such as a patient breaking a hip. The feeling of being in control is key to managing time pressure. However, not only can this experience of lack of time be a barrier for establishing workplace learning, but experiencing a lack of time can cause nurses to omit fundamental conditions important for workplace learning that nurses think are less important, such as good communication [23].

*Cooperation and communication* conditions were both found to be fundamental conditions at the individual, team and organisational levels for workplace learning. Cooperation and communication are conditions which need to be broadly taken into account at every level of the organisation to establish workplace learning. These conditions also facilitate communication with the rest of the organisation, will unite an organisation and therefore create an overall view for an organisation, which are all also important, according to our findings [24]. However, because of the lack of time, nurses will omit these fundamental conditions. Choosing to omit communication actions also occurred within the RN4cast study, where nurses were asked to select actions that were necessary but left undone due to lack of time [23]. Additionally, workload and (lack of) time have been shown to have consequences for residents in the form of fragmented care [25]. This causes the need for more time management, clearly defining necessary actions which cannot be omitted and the need for support to prevent the omission of such actions by, for example, coaching the nursing staff. By creating a learning environment at work, time can actually be saved, as learning at work also means that there is opportunity for ‘just-in-time’ learning. Just-in-time learning means that the learning takes place anywhere and at any time [26]. This gives nursing staff the opportunity to learn directly in practice, with the results of their learning being immediately visible [27].

Other conditions standing out in our results included having an open attitude (*behavioural characteristic*) and (psychological) safety (*context and culture condition*). The ability to feel safe (e.g. to speak up or give/receive feedback) at work and being able to make mistakes and use these as a learning opportunity without severe consequences were key conditions for workplace learning according to our research. Participants mentioned that making mistakes is a part of the learning process at work. Earlier research within a hospital setting reported the same results, where having a climate in an organisation where employees feel safe and mistakes can be made is important for the functioning of teams [28]. Teams that openly report many errors function better at doing their job than teams that do not report errors. Teams that report errors also talk more about (and thus analyse) the errors they encountered, so an open climate prevails and learning opportunities arise naturally. According to Tevlin, Doherty and Traynor (2013), the fear of making mistakes arises from a ‘blame culture’, which can be present in the culture of a healthcare organisation. Looking at long-term elderly care, quality data regarding care are for example only sometimes being used for learning purposes, and are used more for management as external accountability towards third parties who keep track of the quality of care [29]. As a result, nursing staff sometimes become afraid of making mistakes and being accountable. Trust and room for learning and improvement (which includes being able to learn from mistakes) do not benefit from an excessive external accountability to standards set by third parties [30]. Therefore, a shift is needed from a *name, blame and shame* culture to a *no-blame* culture [31]. Within this culture, learning together and learning from mistakes should be possible. Having an open attitude (as an individual but also as a team or organisation) and sense of safety are therefore key conditions for establishing workplace learning in long-term elderly care. To improve this open communication and these (psychological) safety issues, training or coaching programmes can help to overcome these barriers at work and develop a more open and safe working climate [31].

Within community care, a number of social developments are taking place: a shift to ageing in place and more care provision at home, a greater emphasis on clients’ own autonomy, a greater role for informal carers and greater emphasis on collaboration by different care and social workers due to care complexity [32]. Vulnerable elderly living at home often make use of various help and/or care providers [33]. Having multiple different care providers and insufficient information transfer often occurs and this can be a risk indicator for long-term elderly care patients [34]. Compared to a nursing home setting, a different context and culture is present as care professionals in community care work more individually and meet less often with colleagues. Additionally, within community care, the limits to time are strict, as a fixed number of hours are allocated to a client for providing care [35]. Even traveling from one client to the next is charged as working time. This is not the case in nursing home care and makes it harder for care professionals working within community care to cooperate, communicate and learn together, while our research showed that time, cooperation and communication are all important conditions for workplace learning.

Although the results from our research indicated hardly any overall differences between the nursing home and community care setting for necessary conditions for workplace learning, a different approach is necessary because of the different way in which community care is organised. This should include extra attention to the conditions and community care situation mentioned in the paragraph above. It is important to establish time, occasions and opportunities for employees to meet, cooperate and communicate (such as giving and receiving feedback and moments for acquiring knowledge) and to learn together [36]. As team members in community care do not meet each other often, this means for example arranging a clear moment and place for nursing staff to meet and communicate. These meetings can include coaching meetings that vary from organising team (reflection) meetings or debriefings to assignments for acquiring knowledge, as *knowledge and skills* are also key conditions for workplace learning.

Additionally, the current situation around COVID-19 may have accelerated the presence of conditions for establishing workplace learning, because the pandemic was seen as an urgent, exceptional situation. For example, Hung and colleagues showed that there was a sense of increased solidarity between nursing staff to provide the best, safest care possible while also looking out for one another [37]. They also reported an increased level of teamwork as crucial to the nurses’ success. Additionally, nursing staff felt they were well informed and supported by their organisation during the COVID-19 period. Regarding cooperation and communication between team members, nursing staff mentioned that a very good working atmosphere existed during the pandemic [38]. Although COVID-19 also shows negative effects, such as stress and high workload for nursing staff, it does also show that urgency is an important driving factor for improving conditions important for workplace learning.

### Strengths and limitations

It was a strength of the World Café, that a large and heterogeneous group of participants was present and perspectives from different organisations regarding two different settings (nursing home and community care) were taken into account. The findings were also discussed in a plenary session, and a summary of the results was sent as an additional member-check to ensure rigor. We gave participants the opportunity to check the results, to add more information and to check for data saturation. Including table hosts was another strength, as they made sure that new information was discussed in every round. This also facilitated the data saturation of the study. All participants reported their ideas regarding the specific questions of the World Café on a sticky note, which gave everyone the opportunity to explain their thoughts and ensured every opinion was included in the research.

Generalisability of the results may be limited and conclusions need to be drawn with caution due to the specific target group and setting chosen for this research. To get more in-depth information and motives concerning the conditions mentioned in our research, further research – including observations in practice (elderly care) or interviews – is needed to expand the present findings.

## Conclusion

Important conditions to enhance workplace learning within the long-term care setting on the individual, team and organisational levels for nursing staff include facilitating characteristics (e.g. time and room for [team] development), behavioural characteristics (e.g. an open attitude), context and culture (e.g. feeling safe), cooperation and communication (e.g. giving/receiving feedback) and knowledge and skills (e.g. acquiring knowledge from each other). To apply the conditions for workplace learning found in our research, insight into the current learning climate is necessary.

## Data Availability

The datasets generated and/or analysed during the current study are not publicly available due to a language barrier and necessary insight in context for properly understanding the data, but are available from the corresponding author on reasonable request.
